# *N*,*N′*-Bis([1,1′-biphen­yl]-2-yl)-*N*-hy­droxy­methanimidamide

**DOI:** 10.1107/S2414314625008314

**Published:** 2025-09-30

**Authors:** Elizabeth Lamothe, Arindam Saha, Garry S. Hanan, Mihaela Cibian

**Affiliations:** a Département de chimie, Université de Montréal, Complexe des Sciences, 1375 Avenue Thérèse-Lavoie-Roux, Montréal, Québec H2V 0B3, Canada; b Département de biochimie, chimie, physique et science forensique and l’Institut de recherche sur l’hydrogène, Université du Québec à Trois-Rivières. 3351, boul. des Forges, CP 500, Trois-Rivières, Québec, G9A 5H7, Canada; University of Aberdeen, United Kingdom

**Keywords:** crystal structure, amidine *N*-oxide, ligand synthesis

## Abstract

The title compound, C_25_H_20_N_2_O, crystallizes in the monoclinic *C*2/c space group, as the *N*-hy­droxy­formamidine isomer, in the *E* conformation (when referring to the C=N bond). Inversion dimers formed through pairwise O—H⋯N hydrogen bonds are present in the structure. C—H⋯π and π–π stacking inter­actions complete the crystal packing.

## Abstract 2

The title compound displays cyclic dimers formed by O—H⋯N hydrogen bonds, as well as π–π and C—H⋯π stacking inter­actions.

## Graphical abstract

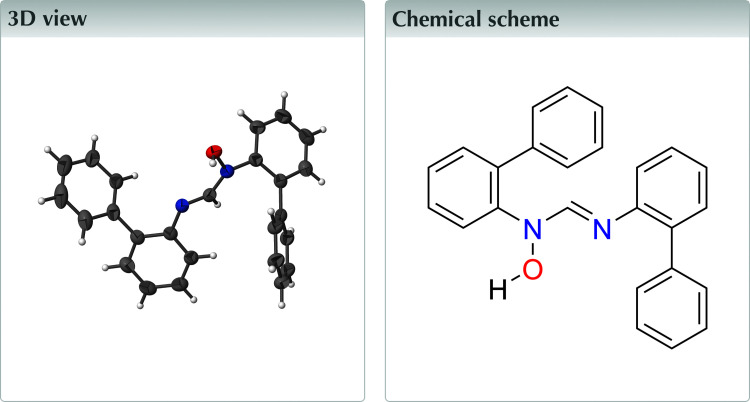

## Structure description

**Figure 1:**
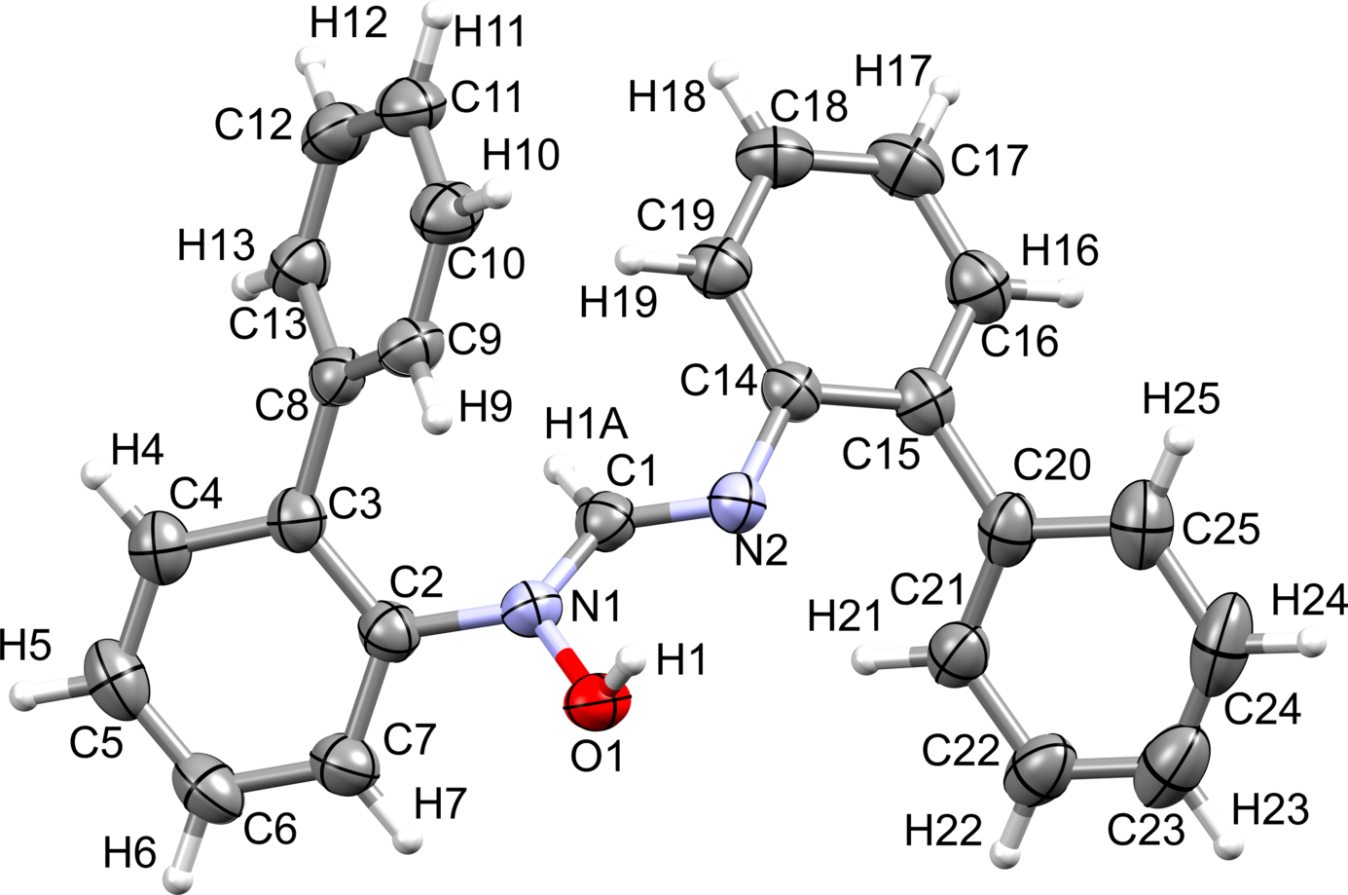
View of the asymmetric unit of **1** with displacement ellipsoids drawn at the 50% probability level.

Colorless XRD-quality single crystals of the title compound, C_25_H_20_N_2_O (**1**), were obtained and intensity data were collected at 100 K. The compound was synthesized as part of a project that explores the coordination chemistry of hy­droxy­amidine/amidine *N*-oxide ligands with transition metal ions to study the structures and properties of the resulting complexes (Verma *et al.*, 1995 ▸; Cibian *et al.*, 2015 ▸; Cibian & Hanan, 2015 ▸; Saha *et al.*, 2024 ▸). Compound **1** crystallizes in the monoclinic *C*2/*c* space group as the *N*-hy­droxy­formamidine isomer, in the *E* conformation (when referring to the C1=N2 bond). The mol­ecule is a symmetrically *N,N′*-disubstituted *N-*hy­droxy­formamidine, consisting of the N2—C1—N1—O1*H* core bearing a peripheral *N*-biphenyl substituent on each of the two N atoms. (Fig. 1 ▸). This is the first report of **1**, but other crystallographic data on *N*-hy­droxy­formamidines/amidine *N-*oxides exist. Free ligands, having symmetrical (Cibian *et al.*, 2009 ▸) and non-symmetrical (Giumanini *et al.*, 1999 ▸) substitution, as well as coordination compounds of cobalt(II) (Cibian *et al.*, 2015 ▸), zinc(II) (Cole *et al.*, 2002 ▸), and copper(II) (Munzeiwa *et al.*, 2021 ▸) have been reported. The bond lengths of the N—C—N—OH bridge in **1** are in line with those already reported for similar compounds crystallized as the *N*-hy­droxy­formamidine isomer form (Cibian *et al.*, 2009 ▸).

In **1**, the bulky 2-biphenyl substituents have tilt angles of 54.0 (1) and 41.2 (1)^ο^ for the C2–C7 and C14–C19 rings, respectively, with respect to the N2–C1–N1 plane. The tilt angles within each of the 2-biphenyl moieties for the C2–C7/C8–C13 and C14–C19/C20–C25 rings are 48.6 (1) and 55.0 (1)^ο^, respectively.

**Figure 2:**
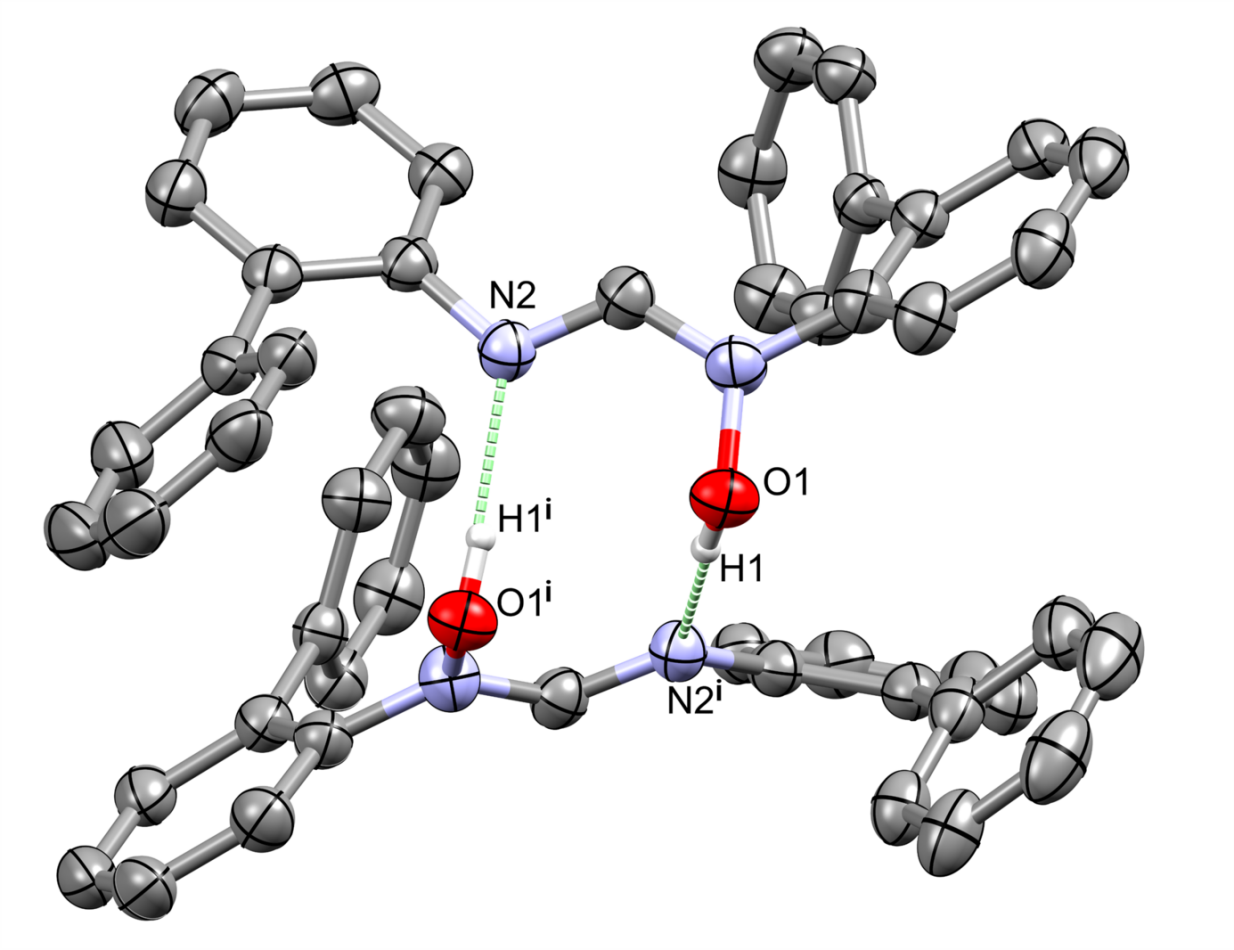
Inter­molecular hydrogen bonding between two mol­ecules of **1** in the unit cell. Symmetry code: (i) −*x* + 1, *y*, −*z* + 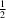.

**Figure 3:**
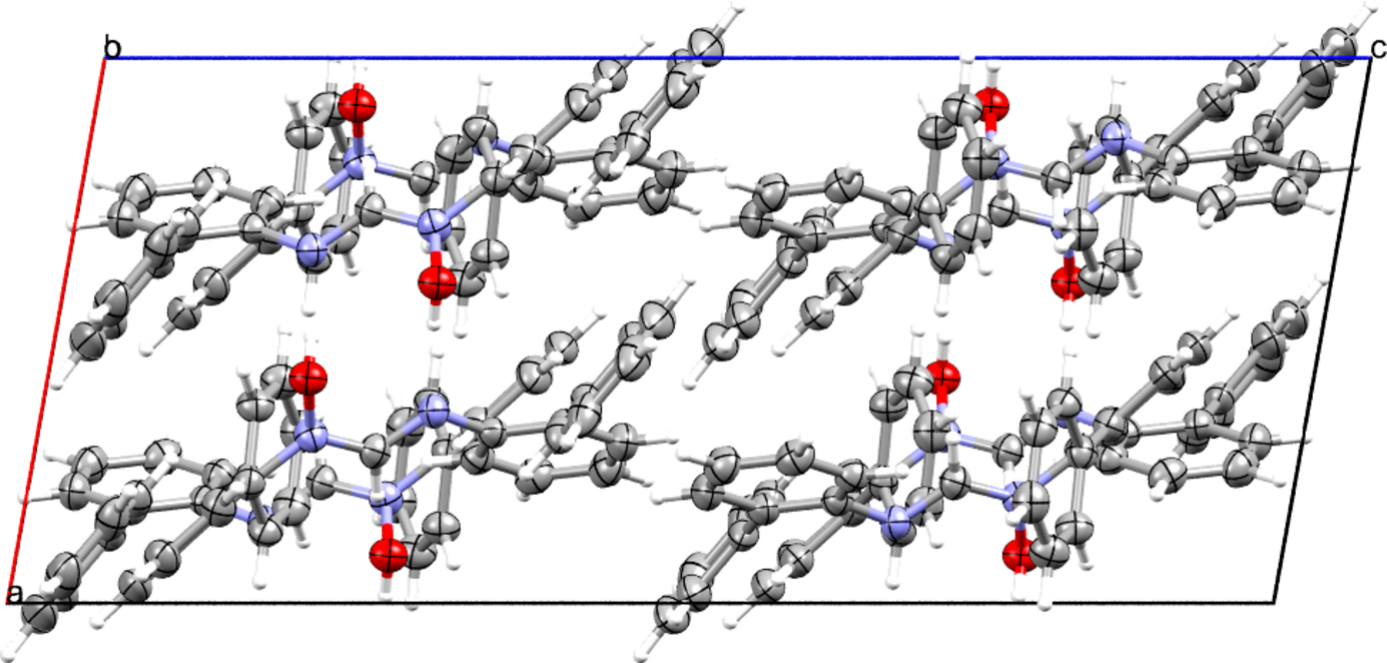
View of the packing of **1** in the unit cell.

Geometric parameters of hydrogen bonds are reported in Table 1 ▸. The structure displays cyclic hydrogen-bonded dimers formed by pairwise O—H⋯N inter­actions (Fig. 2 ▸), which generate 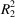
(10) loops. The crystal is efficiently packed (Fig. 3 ▸) by additional C—H⋯O and C—H⋯π inter­actions as well as by multiple π–π stacking inter­actions involving the 2-biphenyl substituents.

## Synthesis and crystallization

Compound **1** was obtained from the oxidation of *N*,*N*′-bis­(2-diphen­yl)formamidine (Cibian *et al.*, 2011 ▸) with *m*-chloro­per­oxy­benzoic acid (*m*-CPBA). *N*,*N′*-bis­(2-diphen­yl)formamidine (1.5 g, 4.3 mmol, 1 equiv.) and NaHCO_3_ (0.38 g, 4.3 mmol, 1equiv.) in DCM (50 mL) were combined with *m*-CPBA (0.96 g, 4.3 mmol, 1 equiv.) in an ice/ methanol bath at −10 °C and stirred for 30–60 minutes, to arrive at room temperature. Liquid–liquid extraction was performed with aqueous K_2_CO_3_ (5%, 2 × 25 mL) and the combined organic layers were dried over anhydrous Na_2_SO_4_. Following filtration and solvent evaporation, a colorless solid was obtained, which was further purified by flash chromatography on silica (gradient of eluents: hexa­ne/EtOAc (2:8), EtOAc/MeOH (9:1), DCM 100%). Recrystallization from a solvent mixture of DCM/hexane (1:1) resulted in colorless plates.

Yield: 0.93 g, 59%. ^1^H-NMR (CDCl3, 400 MHz), p.p.m.: 7.85–7.78 (*m*, 1H, –C_6_**H_4_**), 7.55–7.32 (*m*, 14H, –C_6_**H_5_**, –C_6_**H_4_**, and –NH—C**H**=N–), 7.21 (*dd*, *J* = 7, 2 Hz, 1H, –C_6_**H_4_**), 7.11 (td, *J* = 8, 2 Hz, 1H, –C_6_**H_4_**), 7.05 (*td*, *J* = 7, 1 Hz, 1H, –C_6_**H_4_**), 6.17 (*d*, *J* = 8 Hz, 1H, –C_6_**H_4_**), 3.67 (*bs*, O**H**). ^13^C {^1^H} NMR (CDCl3, 75 MHz) δ, p.p.m.: 142.0, 138.2, 137.5, 136.9, 135.7, 135.2, 131.7, 131.4, 130.7, 129.4 (2 C), 129.24 (2 C), 129.19 (2 C), 129.1 (2 C), 128.8, 128.6, 128.5 (2 C), 128.3, 128.1, 126.1, 123.7, 115.4. Elemental analysis: calculated (%) for C_25_H_20_N_2_O: C 82.39, H 5.53, N 7.69; found: C 82.33, H 5.52, N 7.73. HRMS (ESI positive, DCM) (*m*/*z*): [*M*+H]^+^ C_25_H_21_N_2_O: calculated 365.1648; found 365.1655 (diff. 1.92 p.p.m.)

## Refinement

Crystal data, data collection and structure refinement details are summarized in Table 2 ▸.

## Supplementary Material

Crystal structure: contains datablock(s) I. DOI: 10.1107/S2414314625008314/hb4535sup1.cif

Structure factors: contains datablock(s) I. DOI: 10.1107/S2414314625008314/hb4535Isup2.hkl

Supporting information file. DOI: 10.1107/S2414314625008314/hb4535Isup3.cml

CCDC reference: 2490088

Additional supporting information:  crystallographic information; 3D view; checkCIF report

## Figures and Tables

**Table 1:** Hydrogen-bond geometry (Å, °)

*D*—H⋯*A*	*D*—H	H⋯*A*	*D*⋯*A*	*D*—H⋯*A*
O1—H1⋯N2^i^	0.84	1.91	2.7425 (18)	174
C12—H12⋯O1^ii^	0.95	2.54	3.330 (2)	141
C19—H19⋯*Cg*2	0.95	2.93	3.8724 (19)	173
C22—H22⋯*Cg*2^iii^	0.95	2.89	3.616 (2)	134

Symmetry codes: (i) 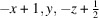; (ii) 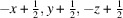; (iii) 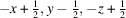.

**Table 2:** Experimental details

Crystal data	
Chemical formula	C_25_H_20_N_2_O
*M*_r_	364.43
Crystal system, space group	Monoclinic, *C*2/*c*
Temperature (K)	100
*a*, *b*, *c* (Å)	10.0344 (4), 16.8774 (6), 22.9241 (9)
β (°)	100.129 (2)
*V* (Å^3^)	3821.8 (3)
*Z*	8
Radiation type	Cu *K*α
μ (mm^−1^)	0.61
Crystal size (mm)	0.18 × 0.10 × 0.05

Data collection	
Diffractometer	Bruker Microstar
Absorption correction	Multi-scan (*SADABS*; Krause *et al.*, 2015 ▸)
*T*_min_, *T*_max_	0.649, 0.753
No. of measured, independent and observed [*I* > 2σ(*I*)] reflections	41862, 3579, 2963
*R*_int_	0.054
(sin θ/λ)_max_ (Å^−1^)	0.608

Refinement	
*R*[*F*^2^ > 2σ(*F*^2^)], *wR*(*F*^2^), *S*	0.046, 0.132, 1.05
No. of reflections	3579
No. of parameters	254
H-atom treatment	H-atom parameters constrained
Δρ_max_, Δρ_min_ (e Å^−3^)	0.24, −0.22

Computer programs: *APEX2* 2 (Bruker, 2009 ▸),and *SAINT* (Bruker, 2009 ▸), *SHELXT* (Sheldrick, 2015*a* ▸), *SHELXL* (Sheldrick, 2015*b* ▸), *OLEX2* (Dolomanov *et al.*, 2009 ▸), *ORTEP-3 for Windows* (Farrugia, 2012 ▸), *publCIF* (Westrip, 2010 ▸), *POVRAY* (POVRAY, 2013 ▸), *PLATON* (Spek, 2020 ▸) and *Mercury* (Macrae *et al.*, 2020 ▸).

## full crystallographic data

### N,N'-Bis([1,1'-biphenyl]-2-yl)-N-hydroxymethanimidamide Crystal data

**Table:**

C_25_H_20_N_2_O	*F*(000) = 1536
*M**_r_* = 364.43	*D*_x_ = 1.267 Mg m^−^^3^
Monoclinic, *C*2/*c*	Cu *K*α radiation, λ = 1.54178 Å
*a* = 10.0344 (4) Å	Cell parameters from 9920 reflections
*b* = 16.8774 (6) Å	θ = 3.9–69.2°
*c* = 22.9241 (9) Å	µ = 0.61 mm^−^^1^
β = 100.129 (2)°	*T* = 100 K
*V* = 3821.8 (3) Å^3^	Plate, colourless
*Z* = 8	0.18 × 0.10 × 0.05 mm

### N,N'-Bis([1,1'-biphenyl]-2-yl)-N-hydroxymethanimidamide Data collection

**Table:**

Bruker Microstar diffractometer	3579 independent reflections
Radiation source: Rotating Anode, Incoatec Iµs	2963 reflections with *I* > 2σ(*I*)
Helios Optics monochromator	*R*_int_ = 0.054
Detector resolution: 8.3 pixels mm^-1^	θ_max_ = 69.6°, θ_min_ = 3.9°
ω scans	*h* = −12→12
Absorption correction: multi-scan (SADABS; Krause *et al.*, 2015)	*k* = −20→14
*T*_min_ = 0.649, *T*_max_ = 0.753	*l* = −27→27
41862 measured reflections	

### N,N'-Bis([1,1'-biphenyl]-2-yl)-N-hydroxymethanimidamide Refinement

**Table:**

Refinement on *F*^2^	Primary atom site location: dual
Least-squares matrix: full	Hydrogen site location: inferred from neighbouring sites
*R*[*F*^2^ > 2σ(*F*^2^)] = 0.046	H-atom parameters constrained
*wR*(*F*^2^) = 0.132	*w* = 1/[σ^2^(*F*_o_^2^) + (0.0645*P*)^2^ + 2.7528*P*] where *P* = (*F*_o_^2^ + 2*F*_c_^2^)/3
*S* = 1.05	(Δ/σ)_max_ < 0.001
3579 reflections	Δρ_max_ = 0.24 e Å^−^^3^
254 parameters	Δρ_min_ = −0.22 e Å^−^^3^
0 restraints	

### N,N'-Bis([1,1'-biphenyl]-2-yl)-N-hydroxymethanimidamide Special details

**Table:**

Experimental. Crystallographic data for the title compound were collected at 150 K, from single crystal samples, which were mounted on a loop fiber. Data were collected using a Bruker Microstar diffractometer equipped with a Platinum 135 CCD Detector, a Helios optics and a Kappa goniometer. The crystal-to-detector distance was 3.8 cm, and the data collection was carried out in 512 x 512 pixel mode. The initial unit cell parameters were determined by a least-squares fit of the angular setting of strong reflections, collected by a 110.0 degree scan in 110 frames over three different parts of the reciprocal space.
Geometry. All esds (except the esd in the dihedral angle between two l.s. planes) are estimated using the full covariance matrix. The cell esds are taken into account individually in the estimation of esds in distances, angles and torsion angles; correlations between esds in cell parameters are only used when they are defined by crystal symmetry. An approximate (isotropic) treatment of cell esds is used for estimating esds involving l.s. planes.
Refinement. The H-atoms were included in calculated positions and treated as riding atoms: aromatic C—H 0.95 Å, methyl C—H 0.98 Å, with *U*_iso_(H) = *k* × *U*_eq_ (parent C-atom), where *k* = 1.2 for the aromatic H-atoms and 1.5 for the methyl H-atoms. The NH proton (H1) was located in the difference-Fourier map and refined freely.

### N,N'-Bis([1,1'-biphenyl]-2-yl)-N-hydroxymethanimidamide Fractional atomic coordinates and isotropic or equivalent isotropic displacement parameters (Å^2^)

**Table:**

	*x*	*y*	*z*	*U*_iso_*/*U*_eq_	
O1	0.41250 (11)	0.32719 (6)	0.29426 (5)	0.0398 (3)	
H1	0.487036	0.350885	0.297375	0.060*	
N1	0.30822 (14)	0.38176 (8)	0.28406 (6)	0.0379 (3)	
N2	0.35221 (14)	0.41337 (8)	0.19030 (6)	0.0361 (3)	
C1	0.27878 (17)	0.41737 (10)	0.23132 (7)	0.0371 (4)	
H1A	0.198039	0.447860	0.223343	0.045*	
C2	0.21963 (17)	0.38428 (10)	0.32616 (7)	0.0379 (4)	
C3	0.17421 (16)	0.45749 (10)	0.34487 (7)	0.0368 (4)	
C4	0.07812 (17)	0.45541 (11)	0.38246 (8)	0.0430 (4)	
H4	0.044016	0.503796	0.395085	0.052*	
C5	0.03203 (19)	0.38461 (13)	0.40153 (8)	0.0498 (5)	
H5	−0.034076	0.384726	0.426541	0.060*	
C6	0.0817 (2)	0.31343 (12)	0.38437 (8)	0.0507 (5)	
H6	0.051431	0.264855	0.398434	0.061*	
C7	0.17569 (19)	0.31302 (11)	0.34661 (8)	0.0453 (4)	
H7	0.209887	0.264218	0.334774	0.054*	
C8	0.22422 (17)	0.53539 (10)	0.32721 (7)	0.0360 (4)	
C9	0.36197 (17)	0.55092 (10)	0.33192 (8)	0.0414 (4)	
H9	0.426106	0.511068	0.346483	0.050*	
C10	0.40633 (19)	0.62414 (10)	0.31553 (9)	0.0469 (4)	
H10	0.500591	0.634216	0.319262	0.056*	
C11	0.3145 (2)	0.68237 (10)	0.29386 (8)	0.0473 (4)	
H11	0.345298	0.732036	0.281965	0.057*	
C12	0.1778 (2)	0.66831 (11)	0.28951 (8)	0.0478 (4)	
H12	0.114173	0.708443	0.274939	0.057*	
C13	0.13330 (18)	0.59558 (10)	0.30641 (8)	0.0418 (4)	
H13	0.038977	0.586569	0.303762	0.050*	
C14	0.31214 (16)	0.46457 (10)	0.14107 (7)	0.0354 (4)	
C15	0.32779 (16)	0.44001 (10)	0.08413 (7)	0.0386 (4)	
C16	0.29510 (19)	0.49359 (12)	0.03720 (8)	0.0478 (4)	
H16	0.305598	0.477661	−0.001484	0.057*	
C17	0.2482 (2)	0.56875 (12)	0.04542 (9)	0.0510 (5)	
H17	0.226304	0.603840	0.012735	0.061*	
C18	0.23304 (19)	0.59280 (11)	0.10161 (9)	0.0470 (4)	
H18	0.199880	0.644356	0.107556	0.056*	
C19	0.26629 (17)	0.54164 (10)	0.14911 (8)	0.0401 (4)	
H19	0.257936	0.558969	0.187735	0.048*	
C20	0.37440 (18)	0.35899 (11)	0.07169 (7)	0.0422 (4)	
C21	0.30844 (19)	0.29159 (11)	0.08680 (8)	0.0444 (4)	
H21	0.236982	0.297108	0.108734	0.053*	
C22	0.3445 (2)	0.21663 (12)	0.07064 (8)	0.0548 (5)	
H22	0.296122	0.171615	0.080504	0.066*	
C23	0.4499 (3)	0.20719 (15)	0.04039 (9)	0.0651 (6)	
H23	0.474305	0.155829	0.028963	0.078*	
C24	0.5199 (2)	0.27266 (17)	0.02675 (10)	0.0678 (7)	
H24	0.595571	0.265921	0.007465	0.081*	
C25	0.4816 (2)	0.34917 (14)	0.04082 (9)	0.0559 (5)	
H25	0.528175	0.394049	0.029472	0.067*	

### N,N'-Bis([1,1'-biphenyl]-2-yl)-N-hydroxymethanimidamide Atomic displacement parameters (Å^2^)

**Table:**

	*U*^11^	*U*^22^	*U*^33^	*U*^12^	*U*^13^	*U*^23^
O1	0.0386 (6)	0.0317 (6)	0.0490 (7)	0.0005 (5)	0.0076 (5)	0.0014 (5)
N1	0.0427 (8)	0.0330 (7)	0.0391 (8)	0.0047 (6)	0.0098 (6)	0.0020 (6)
N2	0.0396 (7)	0.0351 (7)	0.0339 (7)	0.0003 (6)	0.0073 (6)	−0.0008 (6)
C1	0.0410 (9)	0.0340 (8)	0.0361 (8)	−0.0005 (7)	0.0058 (7)	−0.0029 (7)
C2	0.0402 (9)	0.0386 (9)	0.0348 (8)	−0.0035 (7)	0.0062 (7)	0.0000 (7)
C3	0.0360 (8)	0.0411 (9)	0.0327 (8)	−0.0002 (7)	0.0048 (7)	−0.0009 (7)
C4	0.0375 (9)	0.0536 (11)	0.0375 (9)	0.0006 (8)	0.0057 (7)	−0.0008 (8)
C5	0.0421 (10)	0.0689 (13)	0.0391 (10)	−0.0100 (9)	0.0094 (8)	0.0037 (9)
C6	0.0541 (11)	0.0539 (11)	0.0443 (10)	−0.0156 (9)	0.0092 (8)	0.0068 (8)
C7	0.0529 (11)	0.0396 (10)	0.0431 (10)	−0.0061 (8)	0.0076 (8)	0.0030 (7)
C8	0.0406 (9)	0.0348 (8)	0.0334 (8)	0.0022 (7)	0.0081 (7)	−0.0041 (6)
C9	0.0402 (9)	0.0353 (9)	0.0484 (10)	0.0029 (7)	0.0072 (8)	−0.0006 (7)
C10	0.0452 (10)	0.0394 (10)	0.0563 (11)	−0.0052 (8)	0.0098 (8)	−0.0033 (8)
C11	0.0615 (12)	0.0335 (9)	0.0472 (10)	−0.0014 (8)	0.0107 (9)	−0.0004 (7)
C12	0.0566 (11)	0.0377 (9)	0.0483 (10)	0.0112 (8)	0.0073 (8)	0.0003 (8)
C13	0.0408 (9)	0.0425 (9)	0.0418 (9)	0.0059 (7)	0.0063 (7)	−0.0019 (7)
C14	0.0318 (8)	0.0379 (9)	0.0363 (8)	−0.0019 (6)	0.0049 (6)	0.0019 (7)
C15	0.0337 (8)	0.0451 (9)	0.0367 (9)	−0.0018 (7)	0.0052 (7)	0.0015 (7)
C16	0.0488 (10)	0.0557 (11)	0.0386 (10)	0.0005 (9)	0.0074 (8)	0.0052 (8)
C17	0.0540 (11)	0.0518 (11)	0.0448 (10)	−0.0014 (9)	0.0020 (8)	0.0130 (9)
C18	0.0475 (10)	0.0392 (10)	0.0518 (11)	0.0012 (8)	0.0023 (8)	0.0052 (8)
C19	0.0399 (9)	0.0395 (9)	0.0406 (9)	−0.0021 (7)	0.0059 (7)	−0.0005 (7)
C20	0.0399 (9)	0.0541 (11)	0.0315 (8)	0.0098 (8)	0.0033 (7)	−0.0023 (7)
C21	0.0514 (10)	0.0457 (10)	0.0355 (9)	0.0113 (8)	0.0059 (8)	0.0005 (7)
C22	0.0723 (13)	0.0504 (11)	0.0389 (10)	0.0189 (10)	0.0022 (9)	−0.0034 (8)
C23	0.0765 (15)	0.0682 (15)	0.0468 (12)	0.0286 (12)	0.0003 (11)	−0.0116 (10)
C24	0.0560 (13)	0.0987 (19)	0.0494 (12)	0.0254 (13)	0.0111 (10)	−0.0201 (12)
C25	0.0484 (11)	0.0769 (14)	0.0436 (11)	0.0063 (10)	0.0115 (9)	−0.0071 (10)

### N,N'-Bis([1,1'-biphenyl]-2-yl)-N-hydroxymethanimidamide Geometric parameters (Å, º)

**Table:**

O1—H1	0.8400	C12—H12	0.9500
O1—N1	1.3826 (17)	C12—C13	1.384 (3)
N1—C1	1.336 (2)	C13—H13	0.9500
N1—C2	1.423 (2)	C14—C15	1.405 (2)
N2—C1	1.294 (2)	C14—C19	1.403 (2)
N2—C14	1.422 (2)	C15—C16	1.400 (2)
C1—H1A	0.9500	C15—C20	1.489 (2)
C2—C3	1.410 (2)	C16—H16	0.9500
C2—C7	1.390 (2)	C16—C17	1.377 (3)
C3—C4	1.402 (2)	C17—H17	0.9500
C3—C8	1.489 (2)	C17—C18	1.384 (3)
C4—H4	0.9500	C18—H18	0.9500
C4—C5	1.380 (3)	C18—C19	1.384 (3)
C5—H5	0.9500	C19—H19	0.9500
C5—C6	1.384 (3)	C20—C21	1.390 (3)
C6—H6	0.9500	C20—C25	1.398 (3)
C6—C7	1.388 (3)	C21—H21	0.9500
C7—H7	0.9500	C21—C22	1.384 (3)
C8—C9	1.392 (2)	C22—H22	0.9500
C8—C13	1.392 (2)	C22—C23	1.372 (3)
C9—H9	0.9500	C23—H23	0.9500
C9—C10	1.388 (2)	C23—C24	1.375 (4)
C10—H10	0.9500	C24—H24	0.9500
C10—C11	1.379 (3)	C24—C25	1.401 (3)
C11—H11	0.9500	C25—H25	0.9500
C11—C12	1.378 (3)		
			
N1—O1—H1	109.5	C13—C12—H12	120.1
O1—N1—C2	116.72 (13)	C8—C13—H13	119.4
C1—N1—O1	119.54 (13)	C12—C13—C8	121.25 (17)
C1—N1—C2	122.36 (14)	C12—C13—H13	119.4
C1—N2—C14	115.29 (14)	C15—C14—N2	119.73 (15)
N1—C1—H1A	117.6	C19—C14—N2	120.84 (15)
N2—C1—N1	124.78 (16)	C19—C14—C15	119.26 (15)
N2—C1—H1A	117.6	C14—C15—C20	122.64 (15)
C3—C2—N1	120.43 (14)	C16—C15—C14	118.31 (16)
C7—C2—N1	118.41 (15)	C16—C15—C20	119.03 (16)
C7—C2—C3	121.13 (16)	C15—C16—H16	119.0
C2—C3—C8	123.30 (14)	C17—C16—C15	121.97 (17)
C4—C3—C2	117.33 (15)	C17—C16—H16	119.0
C4—C3—C8	119.37 (15)	C16—C17—H17	120.2
C3—C4—H4	119.3	C16—C17—C18	119.57 (17)
C5—C4—C3	121.42 (17)	C18—C17—H17	120.2
C5—C4—H4	119.3	C17—C18—H18	120.0
C4—C5—H5	119.9	C19—C18—C17	119.91 (18)
C4—C5—C6	120.28 (17)	C19—C18—H18	120.0
C6—C5—H5	119.9	C14—C19—H19	119.5
C5—C6—H6	120.0	C18—C19—C14	120.96 (17)
C5—C6—C7	119.98 (17)	C18—C19—H19	119.5
C7—C6—H6	120.0	C21—C20—C15	121.63 (15)
C2—C7—H7	120.1	C21—C20—C25	118.15 (18)
C6—C7—C2	119.77 (17)	C25—C20—C15	120.10 (18)
C6—C7—H7	120.1	C20—C21—H21	119.2
C9—C8—C3	121.52 (15)	C22—C21—C20	121.53 (18)
C9—C8—C13	118.10 (16)	C22—C21—H21	119.2
C13—C8—C3	120.37 (15)	C21—C22—H22	119.9
C8—C9—H9	119.7	C23—C22—C21	120.2 (2)
C10—C9—C8	120.54 (16)	C23—C22—H22	119.9
C10—C9—H9	119.7	C22—C23—H23	120.3
C9—C10—H10	119.8	C22—C23—C24	119.5 (2)
C11—C10—C9	120.42 (17)	C24—C23—H23	120.3
C11—C10—H10	119.8	C23—C24—H24	119.4
C10—C11—H11	120.1	C23—C24—C25	121.1 (2)
C12—C11—C10	119.81 (17)	C25—C24—H24	119.4
C12—C11—H11	120.1	C20—C25—C24	119.5 (2)
C11—C12—H12	120.1	C20—C25—H25	120.2
C11—C12—C13	119.86 (17)	C24—C25—H25	120.2
			
O1—N1—C1—N2	−10.0 (2)	C8—C9—C10—C11	−0.5 (3)
O1—N1—C2—C3	138.89 (15)	C9—C8—C13—C12	1.5 (3)
O1—N1—C2—C7	−43.3 (2)	C9—C10—C11—C12	1.2 (3)
N1—C2—C3—C4	174.64 (15)	C10—C11—C12—C13	−0.6 (3)
N1—C2—C3—C8	−6.0 (2)	C11—C12—C13—C8	−0.8 (3)
N1—C2—C7—C6	−175.36 (16)	C13—C8—C9—C10	−0.8 (3)
N2—C14—C15—C16	−176.18 (15)	C14—N2—C1—N1	−171.55 (15)
N2—C14—C15—C20	5.3 (2)	C14—C15—C16—C17	−0.2 (3)
N2—C14—C19—C18	177.06 (15)	C14—C15—C20—C21	55.2 (2)
C1—N1—C2—C3	−54.6 (2)	C14—C15—C20—C25	−128.83 (19)
C1—N1—C2—C7	123.20 (18)	C15—C14—C19—C18	1.7 (2)
C1—N2—C14—C15	−145.85 (15)	C15—C16—C17—C18	0.3 (3)
C1—N2—C14—C19	38.8 (2)	C15—C20—C21—C22	174.29 (17)
C2—N1—C1—N2	−176.13 (15)	C15—C20—C25—C24	−176.74 (18)
C2—C3—C4—C5	1.4 (3)	C16—C15—C20—C21	−123.26 (19)
C2—C3—C8—C9	−48.9 (2)	C16—C15—C20—C25	52.7 (2)
C2—C3—C8—C13	132.29 (18)	C16—C17—C18—C19	0.6 (3)
C3—C2—C7—C6	2.4 (3)	C17—C18—C19—C14	−1.6 (3)
C3—C4—C5—C6	0.9 (3)	C19—C14—C15—C16	−0.8 (2)
C3—C8—C9—C10	−179.61 (16)	C19—C14—C15—C20	−179.29 (16)
C3—C8—C13—C12	−179.70 (16)	C20—C15—C16—C17	178.35 (17)
C4—C3—C8—C9	130.37 (18)	C20—C21—C22—C23	1.8 (3)
C4—C3—C8—C13	−48.4 (2)	C21—C20—C25—C24	−0.7 (3)
C4—C5—C6—C7	−1.7 (3)	C21—C22—C23—C24	0.5 (3)
C5—C6—C7—C2	0.0 (3)	C22—C23—C24—C25	−2.9 (3)
C7—C2—C3—C4	−3.1 (2)	C23—C24—C25—C20	3.0 (3)
C7—C2—C3—C8	176.24 (16)	C25—C20—C21—C22	−1.7 (3)
C8—C3—C4—C5	−177.91 (16)		

### N,N'-Bis([1,1'-biphenyl]-2-yl)-N-hydroxymethanimidamide Hydrogen-bond geometry (Å, º)

**Table:**

*D*—H···*A*	*D*—H	H···*A*	*D*···*A*	*D*—H···*A*
O1—H1···N2^i^	0.84	1.91	2.7425 (18)	174
C12—H12···O1^ii^	0.95	2.54	3.330 (2)	141
C19—H19···*Cg*2	0.95	2.93	3.8724 (19)	173
C22—H22···*Cg*2^iii^	0.95	2.89	3.616 (2)	134

Symmetry codes: (i) −*x*+1, *y*, −*z*+1/2; (ii) −*x*+1/2, *y*+1/2, −*z*+1/2; (iii) −*x*+1/2, *y*−1/2, −*z*+1/2.
